# Role of early repeated renal biopsies in lupus nephritis

**DOI:** 10.1136/lupus-2014-000018

**Published:** 2014-08-06

**Authors:** A Zickert, B Sundelin, E Svenungsson, I Gunnarsson

**Affiliations:** 1 Department of Medicine, Unit of Rheumatology, Karolinska University Hospital, Karolinska Institute, Stockholm, Sweden; 2 Department of Pathology and Cytology, Karolinska University Hospital, Karolinska Institute, Stockholm,Sweden

**Keywords:** Systemic Lupus Erythematosus, Lupus Nephritis, Autoimmune Diseases

## Abstract

**Objectives:**

A renal biopsy is generally recommended for diagnosis and is necessary for classification of lupus nephritis (LN), but second biopsies after immunosuppressive therapy are seldom a routine procedure. We investigated how repeat biopsies contribute to the evaluation of treatment response and long-term outcome in LN.

**Methods:**

Sixty-seven patients with active LN were included. Renal biopsies were performed at diagnosis and after standard induction immunosuppressive therapy in all patients (median 8 months), regardless of clinical outcome. Biopsies were evaluated according to the International Society of Nephrology/Renal Pathology Society classification. Clinical response was defined as complete (CR), partial (PR) or non-response (NR) according to recent definitions. Histological response (HR) was defined as Class I, II or III/IV-C on repeat biopsies. Long-term renal outcome was determined in 55 patients after a median of 10 years.

**Results:**

CR was demonstrated in 25%, PR in 27% and NR in 48% of patients. HR was shown in 42% and histopathological non-response (HNR) in 58% of patients. Twenty-nine per cent of CR and 61% of patients with PR had active lesions on repeat biopsies, that is, were HNR. Poor long-term renal outcome was associated with high chronicity index at repeated biopsies, but not with clinical or histological response.

**Conclusions:**

Despite apparent clinical response to immunosuppressive therapy, repeated biopsies revealed persisting active nephritis in almost half of the patients, thus providing additional information to clinical response criteria. Repeated renal biopsies may be a tool to improve the evaluation of treatment response in LN.

**Key messages**

- Repeated biopsies revealed persisting active nephritis in many patients, despite clinically low renal disease activity.
- Repeated renal biopsies provide additional information to clinical variables and may be a tool to improve the evaluation of treatment response in LN.

## Introduction

A renal biopsy is the ‘gold standard’ for diagnosis and classification of lupus nephritis (LN), and it is generally agreed that treatment strategies should be based upon histopathological findings.^1–3^ Arguments have been raised that repeat biopsies should be a standard procedure to define response after immunosuppressive treatment, thereby identifying patients who may need prolonged or intensified therapy, but also to avoid overtreatment.^4^ However, there is no consensus on whether a follow-up biopsy should be performed routinely.

It has been shown that patients with LN may have inflammatory activity in the renal tissue without clinical signs of renal involvement or despite good clinical response to therapy.^5^ ^6^ In contrast, in a small study of patients treated with rituximab (RTX), repeated biopsies demonstrated absence of proliferative lesions despite persisting proteinuria.^7^

Biomarkers for renal disease activity in LN are insufficient^8^ and thus, important information may be gained from histopathological findings. However, as a renal biopsy is an invasive procedure with a potential risk for complications, it is important to determine whether a repeat renal biopsy should be performed in all patients with LN or restricted to cases with uncertainties concerning response.

We studied patients with LN in whom repeated biopsies were performed after induction treatment, regardless of clinical response. We compared the histopathological findings with clinical response criteria and studied long-time renal outcome in order to evaluate the contribution of repeated biopsies in LN.

## Methods

### Patients

Sixty-seven patients with biopsy-proven active LN who were followed at the Department of Rheumatology between 1996 and 2009 were included. All patients met at least four of the 1982 American College of Rheumatology classification criteria for systemic lupus erythematosus (SLE).^9^ As a clinical routine at our unit, a second renal biopsy was performed after induction therapy. Clinical data, blood and urinary samples were collected at both biopsy occasions. Patients with a duration of 5 years or more since the baseline biopsy were re-evaluated at last visit in 2011. Informed consent was obtained from all subjects, and the regional ethics committee in Stockholm approved the study protocol.

### Evaluation of renal function and renal disease activity

Renal evaluation included urine analyses (dipslide procedure) and investigation of 24 h urine-albumin excretion. Renal function was determined by serum creatinine (µmol/L) and by estimated glomerular filtration rate (eGFR) using the Modification of Diet in Renal Disease formula.^10^ Chronic kidney disease (CKD) was defined as GFR <60 mL/min.^11^

### Evaluation of renal histopathology

Renal biopsies were evaluated by light microscopy, immunofluorescence and electron microscopy. In 94% of the biopsies, at least 10 glomeruli were available for evaluation and in 8/134 biopsies (6%), 6–9 glomeruli were found. Biopsies were classified according to the International Society of Nephrology/Renal Pathology Society classification,^12^ and scored for activity and chronicity indices.^13^

### Serology and complement measures

Anti-dsDNA antibodies were analysed by multiplex method (Bio-Rad Laboratories, California, USA) according to routine of the laboratory, cut-off <5 IE/mL. The complement components C3 and C4 were determined by nephelometry.

### Definitions of response

Renal response was defined according to a recent consensus statement by Gordon *et al.*^1^ A complete clinical response (CR) was defined as inactive urinary sediment, proteinuria ≤0.2 g/day and normal (GFR >90 mL/min) or stable (within 10% of normal if previously abnormal) renal function. Partial response (PR) was defined by inactive sediment, proteinuria ≤0.5 g/day and normal or stable (<10% deterioration from baseline if previously abnormal) renal function. Patients not reaching these criteria were regarded non-responders (NR).

We also assessed histopathological response (HR); transformation into class I, II or III/IV-C was regarded HR, whereas persistent class III/IV-A or A/C and transformation into class V as histopathological non-response (HNR).

Results were analysed in all patients and also subdivided into patients with proliferative nephritis (PN) (class III or IV) or membranous nephritis (MN) (class V) at baseline biopsies.

### Statistics

We performed the Wilcoxon matched pair test to compare variables at baseline and follow-up. We used the Mann–Whitney test for comparisons between two groups, and the Kruskal–Wallis test for comparisons between multiple groups. For categorical variables, the χ^2^ test was used. Correlations were calculated using Spearman's rank correlation. Statistical significance was set at the level of p<0.05. Statistical evaluation was performed by statistical software, STATISTICA V.9, StatSoft, USA.

## Results

### Outcome at first biopsy

#### Histopathology and renal activity

All patients had an active nephritis at baseline, 57/67 had PN, class III-A or A/C (n=21), IV-A or A/C (n=27), III-IV/V (n=9) and 10/67 had pure MN. Median activity index was 5 (range 0–13) and median chronicity index 1 (range 0–6). The activity index was higher in patients with PN versus MN (p<0.001) while the chronicity index did not differ.

The median creatinine was 84 µmol/L (range 44–284) and median albuminuria 1.4 g/day (range 0–8.4). There was no difference in creatinine or albuminuria in PN versus MN.

#### Serology and complement

Anti-DNA antibodies were detected in 55/57 (96%) of patients, median 165 IE/mL (range 5–300). Levels of C3 were below the reference limit in 70% of patients, median 0.5 g/L (range 0.12–1.13) and C4 were low in 70%, median 0.09 g/L (range 0.02–0.51). Patients with PN had higher anti-DNA (p<0.001), lower C3 and C4 (p=0.02 and 0.03, respectively) compared with MN. The clinical, laboratory and histological characteristics at baseline and follow-up are presented in table 1.

**Table 1:** Clinical, laboratory and histopathological characteristics at first and second biopsies in all patients

	First biopsy	Second biopsy	p Value
Gender, n (%), female	58 (87)		
Male	9 (13)		
Age	34 (18–61)		
Ethnicity, n Caucasian	59		
Hispanic	2		
Asian	3		
African	3		
Creatinine, µmol/L	84 (44–284)	76 (45–306)	*0.003*
Albuminuria, g/day	1.4 (0–8.4)	0.5 (0–3.6)	*<0.001*
C3, g/L	0.5 (0.12–1.13)	0.79 (0.38–1.51)	*<0.001*
C4, g/L	0.09 (0.02–0.51)	0.13 (0.02–0.45)	*<0.001*
Anti-DNA ab IU/mL*	165 (<5–300)	29.5 (<5–300)	*<0.001*
% positive (>5 IU/mL)	96	91	
Renal histology (ISN/RPS), n			
I–II	–	14	
III C	–	13	
III A or A/C	21	10	
IV C	–	1	
IV A or A/C	27	7	
III- IV/V	9	2	
V	10	19	
Vasculitis	–	1	
Activity index	5 (0–13)	2 (0–12)	*<0.001*
Chronicity index	1 (0–6)	1.5 (0–8)	*<0.001*
Prednisolone at biopsy †, %	69	97	
Dose, mg/day	20 (2.5–60)	10 (2.5–60)	*0.003*
Induction treatment, n			
Cyclophosphamide	51		
Mycophenolate mofetil	12		
Rituximab	3‡		
Azathioprin	1§		

All statistical significant p-values (p<0.05) are in italics. Values are presented as median (range) unless otherwise indicated.

*Available in 57 patients.

†% of patients treated with prednisolone.

‡In two patients with class V and 1 with class III.

§In a patient with class V LN.

C3, complement component 3; C4, complement component 4; ISN/RPS, International Society of Nephrology/Renal Pathology Society classification; LN, lupus nephritis.

#### Treatment after first biopsy

The patients were treated with cyclophosphamide (CYC), mycophenolate mophetil (MMF), RTX or azathioprin (AZA) (table 1). The treatment regimen for CYC was 0.5–1 g/m^2^ monthly (modified from the National Institutes of Health protocol),^14^ except for one patient who received low-dose CYC regimen.^15^ All but one (98%) received prednisolone, median 40 mg/day (range 2.5––80), and tapered thereafter. Forty-six patients (69%) were treated with ACE inhibitors and/or angiotensin-II receptor blockers (ARBs).

### Outcome at second biopsy

#### Histopathology and renal activity

Repeated renal biopsies were performed in all patients after a median 8 months (range 5–15) and revealed class I (n=1), class II (n=13), class III-C (n=13), class IV-C (n=1), class III-A or A/C (n=10), class IV-A or A/C (n=7), class III-IV/V (n=2) or class V (n=19). One patient developed a renal vasculitis. Changes in nephritis classification are shown in table 2.

**Table 2:** Changes in nephritis classification (ISN/RPS) from first to second biopsies

Baseline biopsy	Repeated biopsy I/II or III/IV C (n=28)	III/IV A or A/C±V (n=20)	V (n=19)
III A or A/C (=21)	12	8*	1
IV A or A/C (n=27)	14	9	4
V (n=10)	1	0	9
III–IV/V (n=9)	1	3	5

*Including one patient with findings of vasculitis on repeated biopsy.

A, with active lesions; A/C, with both active and chronic lesions; C, with chronic lesions; ISN/RPS, International Society of Nephrology/Renal Pathology Society classification.

In all patients and in PN, the renal activity index decreased (p<0.001), whereas there was an increase in chronicity index (p<0.001), but no significant change was seen in MN.

In all patients and in PN, creatinine decreased (p=0.003 and 0.004, respectively), however, not in MN. Overall, albuminuria decreased (p<0.001), significant both in patients with baseline PN and MN (p<0.001 and p=0.04, respectively) (see table 1 for all patients).

#### Serology and complement

In all patients, anti-DNA decreased between the biopsies (p<0.001), but remained positive in 91% of patients. In patients with baseline PN, anti-DNA decreased significantly (p<0.001), which was not found in MN (p=0.09). In all patients, C3 and C4 increased (p<0.001 for both) (table 1), but remained low in 36% and 34%, respectively.

#### Clinical and histopathological response

A clinical response (CR or PR) was observed in 35/67 (52%) patients at the time of repeated biopsies. Seventeen patients had CR (25%), 18 had PR (27%) and 32 had NR (48%).

Patients with PN had 17 CR, 15 PR and 25 NR, whereas patients with MN had no CR, 3 PR and 7 NR.

No differences in creatinine, C3, C4 or anti-DNA were found at either baseline or follow-up in NR versus CR/PR. Laboratory and histopathological data at the time of repeat biopsies in association to clinical response are presented in table 3.

**Table 3:** Laboratory and histopathological characteristics at the time for repeat biopsy in clinical complete, partial and non-responders (all patients)

	CR (n=17)	PR (n=18)	NR (n=32)	p Value
Anti-DNA IU/mL*	34 (10–120)	22 (7–250)	45 (5–300)	0.67
C3, g/L	0.81 (0.38–1.31)	0.93 (0.6–1.38)	0.72 (0.45–1.51)	0.28
C4, g/L	0.12 (0.06–0.28)	0.16 (0.1–0.45)	0.13 (0.02–0.29)	0.08
Renal histology†, n				
Class I–II	6	4	4	
Class III/IV (C)	6	3	5	
Class III/IV (A) or (A/C)	1	7	10	
Class III/IV (A)+V	0	0	2	
Class V	4	4	11	
Activity index	1 (0–3)	2 (1–9)	2 (0–12)	*0*.*001*
Chronicity index	1 (0–6)	3 (0–8)	1.5 (0–8)	0.26
Histological response				
HR, n (%)	12 (71)	7 (39)	9 (28)	
HNR, n (%)	5 (29)	11 (61)‡	23 (72)	

All statistical significant p-values (p<0.05) are in italics. Values are presented as median (range) unless otherwise indicated.

*Available in 57 patients.

†According to the International Society of Nephrology/ Renal Pathology Society (ISN/RPS) classification.

‡Including the patient with a renal vasculitis on the repeat biopsy.

A, with active lesions; A/C, with both active and chronic lesions; C, with chronic lesions; C3, complement component 3; C4, complement component 4; CR, complete response; HNR, histopathological non-response; HR, histopathological response; n, number of patients; NR, non-response; PR, partial response.

Overall, HR was seen in 28/67 (42%) and HNR in 39/67 (58%) patients. In HNR patients, second biopsies revealed PN or mixed PN/MN (n=19), MN (n=19) and one renal vasculitis.

In patients with baseline PN, 27/57 (47%) had HR, and 30/57 (53%) had HNR of which 20/30 had persisting PN or PN/MN and 10/30 had transformed into MN. Of patients with baseline MN, only one changed nephritis class (to class III-C).

There was no difference in either clinical or HR with respect to the induction treatment given.

### Factors predicting histopathological outcome, associations between clinical and histopathological response

Of the 17 patients with CR (all PN), 29% were HNR and of the 18 patients with PR (15 PN and 3 MN), 61% were HNR. In contrast, 28% with poor clinical response (NR) had no active lesions on repeat biopsies (HR) (table 3). Patients with CR had lower activity indices on repeat biopsies versus PR and NR (p=0.001).

Of patients with baseline PN with PR, 8/15 (60%) were HNR. In contrast, 8/25 (32%) patients with clinical poor response (NR) had a good histological response (ie, HR) (table 4).

**Table 4:** The number of patients with histopathological response (HR) and non-response (HNR) in relation to clinical complete (CR), partial (PR) and non-response (NR) in patients with (A) baseline proliferative nephritis (PN) and (B) baseline membranous nephritis (MN)

(A) PN	CR (n=17)	PR (n=15)	NR (n=25)
HR	12	7	8
HNR	5	8	17
(B) MN	CR (n=0)	PR (n=3)	NR (n=7)
HR	0	0	1
HNR	0	3	6

At repeat biopsies, renal activity index correlated to albuminuria (r=0.4, p<0.05) and anti-DNA (r=0.4, p<0.05). Chronicity index correlated to creatinine (r=0.3, p<0.05) (data not shown).

Overall, HNR patients had higher levels of proteinuria at follow-up versus HR (p=0.003), also found when analysing PN separately (p=0.006). Of the 23 patients with albuminuria 0.2 g/day or less at follow-up, 8 (35%) had persisting active nephritis (HNR) at repeated biopsy (3 PN and 5 MN) and of 37 patients with albuminuria 0.5 g/day or less, 18 (49%) were HNR.

In all patients, levels of anti-DNA at follow-up did not differ between HR and HNR, but in patients with baseline PN, anti-DNA were higher in HNR versus HR (p=0.05). Clinical data at repeat biopsies in PN patients with HR versus HNR are presented in table 5.

**Table 5:** Laboratory findings at the time for repeat biopsy and clinical response in patients with baseline PN in histopathological responders vs non-responders

	HR (n=27)	HNR (n=30)	p Value
Creatinine, µmol/L	74 (48–306)	76 (45–122)	0.88
Albuminuria, g/day	0.2 (0–3.4)	0.6 (0.03–3.1)	*0.007*
Anti-DNA IU/mL*	18 (<5–250)	49 (<5–300)	*0.03*
C3, g/L	0.74 (0.4–1.38)	0.81 (0.38–1.51)	0.83
C4, g/L	0.13 (0.04–0.3)	0.13 (0.03–0.45)	0.53
Hematuria†, %	30	54	0.11
Clinical response, n			
CR	12	5	
PR	7	8	
NR	8	17	

All statistical significant p-values (p<0.05) are in italics. Values are presented as median (range) unless otherwise indicated.

*Available in 47 patients.

†u-erytrocytes >1+ in % of patients.

C3, complement component 3; C4, complement component 4; CR, complete response; HNR, histopathological non-response; HR, histopathological response; n, number of patients; NR, non-response; PN, proliferative nephritis; PR, partial response.

#### Treatment after repeat biopsies

The treating clinician decided treatment strategy after the second biopsy. This included prolonged treatment with CYC (n= 10), switching from CYC to AZA (n=26), from CYC to MMF (n= 6), prolonged MMF (n=11), switching from CYC/MMF to RTX (n=6) or no further immunosuppressive treatment (n=2). The patients initially treated with RTX received AZA (n=1), MMF (n=1) and one had no immunosuppressive treatment after second biopsy. The patient initially treated with AZA received MMF.

Sixteen patients with HNR were clinical responders, CR (n=5) or PR (n=11) (table 3). Of these, 13 (81%) received prolonged or intensified treatment with CYC/MMF or were switched to RTX. Of the 13 PN patients with HNR despite CR/PR, 12 (92%) received prolonged or intensified treatment.

### Outcome at long-term follow-up

At last follow-up, three patients had died, and two were lost to follow-up. Of the three patients that died, all had a follow-up period of more than 5 years. In all, 56 patients (mean age 43 years, range 26–72) had a duration of 5 years or more since baseline biopsy (median 10 years, range 5–15) and were available for long-term follow-up. Of the 56 patients, four were in end-stage renal disease (ESRD), (including one of the patients that had died).

One patient had severely impaired renal function already at the baseline biopsy and was excluded from the analyses, thus a total of 3/55 (5%) developed ESRD during the observation time. In the 52 non-ESRD patients, median creatinine was 72.5 (range 36–186) and median eGFR 80 (31–160). Altogether (including ESRD patients) 14/55 (25%) of the patients had an estimated GFR <60 mL/min, median 52 (range 31–59), and was regarded as having CKD^11^. Except ESRD patients, none developed a doubling of serum creatinine.

Forty-eight per cent of the patients had ongoing immunosuppressive therapy (AZA or MMF), 42% had antimalarials, 74% had prednisolone (median dose 5 mg, range 1.25–25 mg) and 63% were treated with ACE-inhibitors or ARBs. Additionally 14 of the patients had been treated with RTX since the second biopsy due to either persisting (ie, lack of response) or relapse of nephritis.

### Clinical and histological findings at first and second biopsies, association to renal outcome

Overall, creatinine at long-term follow-up correlated to creatinine at both baseline and follow-up biopsies (r=0.5, p<0.05 for both), but no correlations to anti-DNA, proteinuria, C3 or C4 at either baseline or follow-up biopsies were documented.

Patients who developed CKD (ESRD or eGFR<60, n=14) had significantly higher chronicity index on repeated biopsies (p=0.03), but no difference was found for activity index. Overall, patients with CKD had higher creatinine at repeat biopsies (p<0.01). Laboratory and histological findings at first and second biopsies in patients with eGFR >60 vs eGFR<60 at long-term follow-up are presented in table 6. There was no difference in renal function at long-term follow-up with respect to the induction treatment given after baseline biopsies.

**Table 6:** Laboratory and histological findings at first and second biopsies in patients with eGFR>60 vs. eGFR<60 at long-term evaluation (median 10 years)

	eGFR>60, n=41		eGFR<60, n=14		
	1st biopsy	2nd biopsy	1st biopsy	2nd biopsy	p Value
Creatinine, µmol/l	82 (52–185)	75 (45–95)	105 (52–176)	86 (66–128)	ns *<0.01*
Albuminuria, g/d	1.4 (0.1–7.4)	0.7 (0–3.4)	2.2 (0–8.1)	0.5 (0–3.6)	ns ns
Anti–DNA, IU/ml*	200 (8–300)	26.5 (<5–300)	161 (13–300)	16 (<5–170)	ns ns
C3, g/l	0.44 (0.12–0.94)	0.78 (0.4–1.41)	0.5 (0.3–0.8)	0.9 (0.38–1.51)	ns ns
C4, g/l	0.07 (0.02–0.24)	0.13 (0.03–0.3)	0.11 (0.03–0.16)	0.14 (0.04–0.26)	ns ns
Clinical response					
CR, n		11		3	ns
PR, n		9		4	ns
NR, n		16		6	ns
Nephritis class^†^, n					
I/II/III C		16		5	ns
III/IV A-A/C	36	13	11	5	ns
V	6	12	3	3	ns
HR, n		16		6	ns
HNR, n		25		8	ns
Activity index	5 (0–13)	2 (0–10)	5 (1–13)	1.5 (0–12)	ns ns
Chronicity index	1 (0–6)	1 (0–8)	2 (0–6)	3.5 (0–7)	ns *0.03*

Values are presented as median (range) unless otherwise indicated.

*Available in 47 patients.

†According to the International Society of Nephrology/Renal Pathology Society (ISN/RPS) classification.

C3, complement component 3; C4, complement component 4; CR, complete response; eGFR, estimated glomerular filtration rate; HNR, histopathological non-response.HR, histopathological response, NR, non-response; PR, partial response; ns, non-significant.

All the three patients who developed ESRD had PN at baseline and all were HNR with both active and chronic lesions at follow-up biopsies (two III-A/C and one class V). ESRD patients had lower C4 (p=0.05) and higher anti-DNA (p=0.04) at repeat biopsies versus non-ESRD patients. There was no significant difference in creatinine, proteinuria or C3 at baseline or follow-up biopsies in ESRD versus non-ESRD patients (data not shown).

## Discussion

This is a large study on repeated renal biopsies, here performed consecutively and regardless of clinical response, after immunosuppressive induction treatment in patients with LN. We demonstrate a persistent histopathological renal activity despite apparent clinical quiescent disease in a substantial proportion of patients. In contrast, no active inflammatory lesions in repeat biopsies were present in a group of patients with clinically poor therapeutic response. The clinical and histopathological findings were thus highly discordant, as also indicated in previous studies.^5^ ^6^ ^16^

Only a limited number of studies have previously evaluated the value of repeated biopsies in LN, and as timing of the biopsies, the selection of the patients and the size of the studies differ, it is difficult to fully compare the results. However, some previous studies reported that findings on second biopsies were predictive of long-term prognosis. In the early study by Esdaile *et al*,^17^ decreased amounts of immune deposits on repeat biopsies performed after a median of 25 months were found to be the best predictor for a favourable renal outcome. Consistent with our study, Hill *et al*^18^ performed repeat renal biopsies in patients with LN after 6 months of treatment and demonstrated a discordance between clinical and histopathological findings. In contrast to our data, they reported that active inflammatory lesions on repeat biopsies predicted poor long-term renal outcome. In line with our findings, Moroni *et al*^19^ demonstrated that high chronicity indices on repeat biopsies predicted a poor renal outcome. However in that study, biopsies were performed at later stages and in selected patients with signs of more severe renal disease, which may have influenced the results. A recent study on second kidney biopsies performed after 12–18 months demonstrated that histopathological evidence of active disease at repeat biopsy, regardless of clinical response, was predictive of poor renal survival after a median of 8.7 years.^20^ As the study population was not restricted to active patients with LN only, our studies are not fully comparable. In a study of PN, comprising patients randomised to AZA or CYC, repeated biopsies after 2 years did not provide any additional information regarding the long-term renal outcome.^21^ Less than half of the study population underwent a repeat biopsy in that study, however, which may have influenced the results. Our findings of discordance between histopathological findings and the laboratory variables routinely used to define disease activity, and also the prognostic information obtained from repeat renal biopsies, emphasise that it is important to further evaluate the impact of histopathological versus clinical response in LN.

Proteinuria is commonly used as a biomarker for activity in LN and, in accordance with previous data,^6^ we found that HNR had more pronounced proteinuria than HR. On the other hand, almost half of the patients with low-grade proteinuria had active lesions in the renal tissue at repeated biopsy. Furthermore, of patients with baseline PN, 30% had active nephritis on repeat biopsies despite having a CR.

Anti-DNA are among the most specific antibodies in lupus, repeatedly found to associate with disease activity.^22^ ^23^ Although the levels of anti-DNA antibodies correlated to activity indices at repeat biopsies, and patients with HNR were shown to have higher levels of anti-DNA, a proportion of patients with persisting active nephritis still had very low anti-DNA levels. Taken together, our findings demonstrate that clinical parameters, including albuminuria, levels of anti-DNA antibodies and complement levels, which are all commonly used for evaluation of renal response, are not reliable biomarkers in LN.

There are no generally used response criteria available in LN, which is illustrated by the differences in response criteria used in clinical trials.^15^ ^24^ ^25^ This is an issue of concern as differences in definitions of response may lead to diverging results in clinical trials. This was recently demonstrated in a study by Wofsy *et al*,^26^ where the effect of abatacept in patients with LN differed substantially depending on which response criteria were used. Our data suggest that repeated biopsies may contribute to an improvement in the evaluation of renal response. Given the problems to define clinical response criteria, and also in the light of unsuccessful clinical trials in LN, it has been suggested that second renal biopsies should be considered in the study design of future clinical trials.^27^ Of note, repeat renal biopsies can distinguish between active LN and chronic lesions but can also identify vascular lesions (ie, thrombotic microangiopathy), which may all present clinically with persisting or increasing proteinuria or rising creatinine but should be treated differently.

Since most patients with MN have persisting proteinuria over a longer period of time, response is even more difficult to define. This was further illustrated in our study as no patients with MN experienced a CR and only a few had PR. In a recent study on patients with MN treated with RTX, electron microscopy revealed resorption of subepithelial immune deposits in repeated biopsies in parallel with clinical response.^28^ Thus, electron microscopy evaluation of immune deposits may be an additional tool to analyse histological response, as also shown by Esdaile *et al,*^17^ and needs to be further studied in MN.

There are currently no consensus definitions for HR in LN, and HR has, to date, not been included in clinical trials. In the current study, we defined the presence of persistent active nephritis, that is, class III or IV A-A/C or V, in repeat renal biopsies as a HNR. It could be an issue of discussion if a class V should also be regarded as HR or not. However, class V represents an active nephritis with need of therapeutic interventions and was here regarded as a non-responder.

The ultimate goal for LN treatment is to preserve renal function over time. Although the mortality of patients with SLE has dramatically improved, renal survival in LN over the past few decades remains unchanged.^29^ It is well known that impaired renal function is associated with cardiovascular disease and increased mortality in the general population,^30^ and also in patients with SLE.^31^ Identification of patients at risk for developing CKD is a major concern and previous studies have demonstrated that early response to treatment can predict long-term outcome.^32^ Second biopsy findings may identify patients who could benefit from intensified immunosuppressive treatment in early phase and may thus limit the development of irreversible damage and improve long-term renal outcome in LN.

In our study, 5% developed ESRD and no patient had a doubling of creatinine. Although no firm conclusions can be drawn, the good long-term outcome of our patients as compared with other studies^18^ ^20^ ^29^ ^33^ could at least partially be due to the intense treatment given to histopathological NR. As non-Caucasians have been shown to have more severe LN,^29^ the mainly Caucasian ethnicity of our patients may also have contributed to the beneficial outcome. Many patients also received more intense treatment, including rituximab, later in the disease course, which may also have an impact on long-term outcome.

In order to determine the predictive value of histological findings on repeated renal biopsies, the most appropriate approach would be to perform a randomised trial in which patients with persisting or increasing histological activity are randomly assigned to intensified immunosuppressive therapy or traditional maintenance therapy. Our study provides important baseline knowledge for such a trial. However, ethical considerations may influence the performance of the suggested study.

This is a retrospective and observational ‘real life’ study, associated with several limitations that impede interpretation of the results for long-term outcome. Multiple factors such as different treatment regimens, variations in doses of prednisolone, control of blood pressure and other comorbidities as well as patient compliance may have impact on the results. Treatment strategies have changed over time and currently the low-dose CYC (Euro-lupus) regimen^15^ or MMF is standard of care for LN. In addition, the increasing use of ACE-inhibitors/ARB and more patients on background treatment with antimalarials may probably influence long-term renal outcome.^34^

In this study, 24 h albuminuria was measured while many studies on LN have used total proteinuria for evaluation of response. Although proteinuria and albuminuria might yield comparable results, a nonlinear relationship between albuminuria and total proteinuria has been described in patients with very low grade proteinuria. In that situation, albuminuria is proportionately a smaller fraction of the total proteinuria,^35^ which here may have had impact on the results regarding clinical response. For future studies on LN, measurement of total proteinuria should be considered as an outcome measure.

To conclude, we report that many patients with LN had active lesions in repeat renal biopsies after immunosuppressive treatment despite clinically low renal disease activity. Histological evaluation provides additional information that is not captured by routine laboratory variables. An RCT is needed to test whether repeated biopsies should be considered as a part of the evaluation of treatment response in LN.
